# Eukaryotic initiation factor 5A2 mediates hypoxia-induced autophagy and cisplatin resistance

**DOI:** 10.1038/s41419-022-05033-y

**Published:** 2022-08-05

**Authors:** Guodong Xu, Hang Chen, Shibo Wu, Jiabin Chen, Shufen Zhang, Guofeng Shao, Lebo Sun, Yinyu Mu, Kaitai Liu, Qiaoling Pan, Ni Li, Xiaoxia An, Shuang Lin, Wei Chen

**Affiliations:** 1 Department of Cardiothoracic Surgery, the Affiliated Lihuili Hospital, Ningbo University, Ningbo, PR China; 2 Medical School, Ningbo University, Ningbo, PR China; 3 Department of Oncology, Tongde Hospital of Zhejiang Province, Hangzhou, PR China; 4 Cancer Institute of Integrated Traditional Chinese and Western Medicine, Zhejiang Academy of Traditional Chinese Medicine, Tongde Hospital of Zhejiang Province, Hangzhou, PR China; 5 Department of Anesthesiology, the First Affiliated Hospital, College of Medicine, Zhejiang University, Hangzhou, Zhejiang China; 6 Department of Thoracic Surgery, the First Affiliated Hospital, College of Medicine, Zhejiang University, Hangzhou, Zhejiang China

**Keywords:** Cell biology, Lung cancer

## Abstract

Hypoxia-induced cisplatin resistance is a major challenge during non-small cell lung cancer (NSCLC) treatment. Based on previous studies, we further explored the effect of eukaryotic initiation factor 5A2 (eIF5A2) in hypoxia-induced cisplatin resistance. In this study, we found that autophagy and cisplatin resistance were increased under hypoxic conditions in three different NSCLC cell lines. Compared with that under normoxic conditions, dramatic upregulation of eIF5A2 and hypoxia inducible factor 1 subunit alpha (HIF-1α) levels were detected under hypoxia exposure. Small interfering RNA silencing of HIF-1α resulted in decreased expression of eIF5A2, indicating that eIF5A2 acts downstream of HIF-1α. In addition, the expression of eIF5A2 was significantly higher in NSCLC tumors compared with that in normal tissues. RNA silencing-mediated downregulation of eIF5A2 decreased hypoxia-induced autophagy, thereby reducing hypoxia-induced cisplatin resistance in NSCLC cells. The roles of eIF5A2 in cisplatin resistance were further validated in vivo. Combined treatment using eIF5A2-targeted downregulation together with cisplatin significantly inhibited tumor growth compared with cisplatin alone in the subcutaneous mouse model. In conclusions, eIF5A2 overexpression is involved in hypoxia-induced autophagy during cisplatin resistance. We suggest that a combination of eIF5A2 targeted therapy and cisplatin chemotherapy is probably an effective strategy to reverse hypoxia-induced cisplatin resistance and inhibit NSCLC development.

## Introduction

Lung cancer is the second most common cancer and the leading cause of death for both male and female cancer patients in the USA and China [1, 2]. Approximately 85% of lung cancer is non-small cell lung cancer (NSCLC), of which more than half are lung adenocarcinoma (LUAD) [3]. The 5-year overall survival (OS) of NSCLC with distant metastasis is only 7%, because it is always diagnosed at an advanced stage and conventional therapy is ineffective [4]. Cisplatin, a platinum-based chemotherapeutic drug routinely used drug to treat advanced NSCLC, has limited efficacy because of the development of cisplatin resistance [5]. Therefore, understanding the molecular mechanisms underlying cisplatin resistance and identifying novel and effective targets are important for the clinical treatment of advanced NSCLC.

The microenvironment of solid tumors, such as NSCLC and hepatocellular carcinoma, suffers from chronic hypoxia [6]. Local tumor hypoxia is the leading cause of chemotherapy resistance, and hypoxia inducible factor-1α (HIF-1α) is the main hypoxia-induced transcription factor regulating the expression of chemoresistance-related genes [7, 8]. HIF-1α-regulated genes promote blood vessel formation, red blood cell production, sugar metabolism, and regulate cell apoptosis to adapt to hypoxic conditions [9, 10]. Hypoxia also induces eIF5A2 (encoding eukaryotic translation initiation factor 5 A) expression, which is an oncogene located at 3q26 associated with progression in various cancers, including NSCLC, bladder cancer, and colorectal cancer (CRC) [11–13]. EIF5A2 also induces autophagy, leading to chemotherapy resistance in various cancers. For example, reduced expression of eIF5A2 inhibited autophagy and enhanced apoptosis, which significantly improved doxorubicin sensitivity [14]. A recent report suggested that eIF5A2 plays roles in chemotherapy resistance in NSCLC [15]. Our previous research [16, 17] showed that eIF5A2 enhanced NSCLC cell cisplatin resistance by regulating epithelial-mesenchymal transition (EMT) and modulating NSCLC cell invasion and migration. In addition, silencing eIF5A2 markedly reduced cetuximab resistance in NSCLC [15]. Hypoxia-induced autophagy has been suggested to mediate NSCLC chemoresistance [18]; therefore, we hypothesized that eIF5A2 might promote NSCLC chemotherapy resistance by enhancing autophagy. In this study, we found that eIF5A2 expression was significantly enhanced in NCLC tumors. In addition, downregulating eIF5A2 expression using a small interfering RNA (siRNA) significantly increased the expression of autophagy-related genes (LC3 (encoding microtubule associated protein 1 light chain 3), P62 (also known as SQSTM1, encoding sequestosome 1), BECN1 (encoding beclin1, and ATG3 (encoding autophagy related 3)) and reduced hypoxia-induced cisplatin resistance in A549 cells. Furthermore, an in vivo subcutaneous model showed that a combination of eIF5A2 downregulation and cisplatin markedly inhibited tumor growth compared with cisplatin treatment alone. Taken together, our results suggested that eIF5A2 is a promising target to overcome hypoxia-induced chemoresistance in NSCLC, and cisplatin chemotherapy combined with eIF5A2 targeted therapy could effectively hinder NSCLC development, which will benefit patients with advanced or relapsed NSCLC who develop chemotherapy resistance.

## Results

### Hypoxia increases cisplatin resistance and autophagy of NSCLC cells

**Fig. 1:**
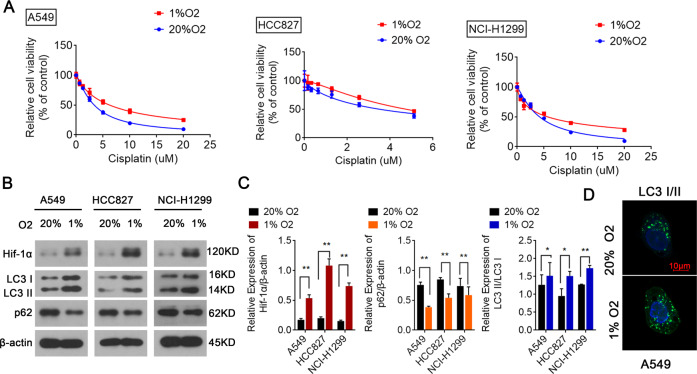
Enhanced autophagy and cisplatin resistance was observed in NSCLC cells under hypoxic conditions. **A** CCK-8 assays suggesting that the viability of A549, HCC827, and NCI-H1299 cells treated with cisplatin increased after hypoxia exposure. **B**, **C** Western blotting indicating that LC3II/LC3I and HIF-1α levels increased and p62 levels decreased in three NSCLC cell lines (A549, HCC827, and NCI-H1299) under hypoxic conditions. **D** Staining for LC3 (green) and DAPI nuclear staining (blue) in A549 cells, showing that LC3II/LC3I expression increased in A549 cells under hypoxic conditions, and LC3 was located in the cytoplasm. *P < 0.05, **P < 0.01.

Hypoxia-induced autophagy might contribute to NSCLC chemoresistance [19]; however, that study only used A549 and SPC-A1 cells. Here, we explored the role of hypoxia in cisplatin resistance in three NSCLC cell lines: A549, HCC827, and NCI-H1299. To investigate whether the cisplatin sensitivity is statistically significant under hypoxia and normoxia conditions, we performed cell counting kit 8 (CCK8) assays, which indicated that the cisplatin sensitivity of all three NSCLC cell lines decreased markedly after exposure to 1% O_2_ compared with exposure to 20% O_2_, suggesting significant hypoxia-induced cisplatin resistance (Fig. 1A). Next, the investigation of the relationship between the autophagy-related genes (e.g., LC3 and p62) and hypoxia showed that hypoxia exposure increased LC3II/LC3I and HIF-1α levels, but decreased p62 levels in all three NSCLC cell lines, suggesting that hypoxia could induce autophagy (Fig. 1B, C). The increased levels of LC3II/LC3I in A549 cells under hypoxic conditions was confirmed using immunofluorescence microscopy (Fig. 1D). Increased LC3 puncta were observed in the cytoplasm of hypoxia-treated A549 cells (Fig. 1D). Our findings are consistent with previous reports that hypoxia-induced autophagy is related to NSCLC cisplatin resistance.

### HIF-1α mediates eIF5A2 overexpression under hypoxia

**Fig. 2:**
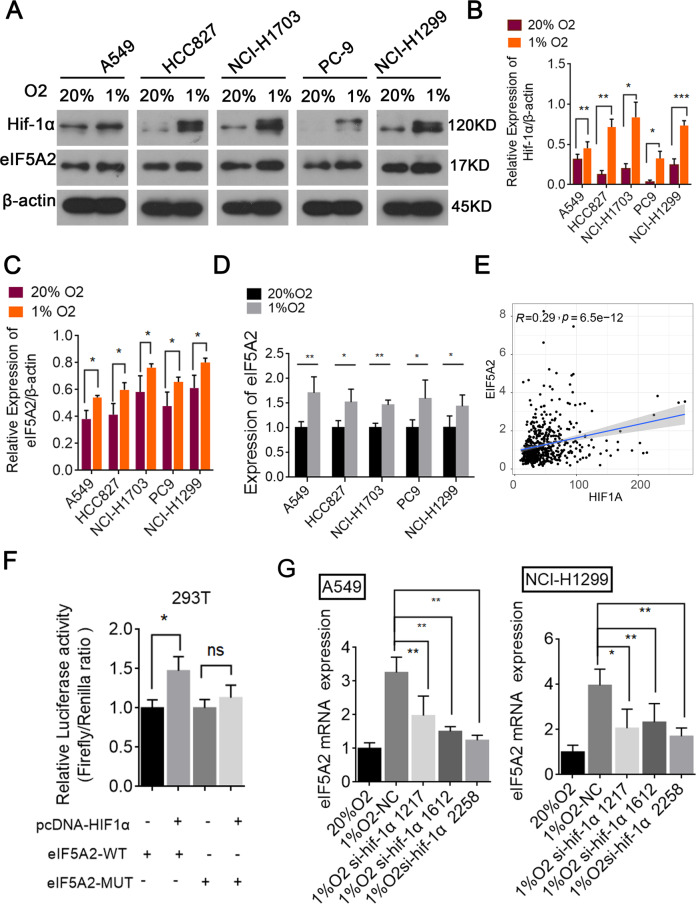
Expression of HIF-1α and eIF5A2 were upregulated under hypoxic conditions. **A**–**D** Western blotting (**A**–**C**) and qRT-PCR (**D**) results showing that the expression levels of HIF-1α and eIF5A2 in five NSCLC cell lines (A549, HCC827, NCI-H1703, PC9, and NCI-H1299) increased under hypoxic conditions. **E** A scatter plot suggesting that eIF5A2 levels correlated significantly and positively with *ATG3* levels (*R* = 0.36, *p* < 0.001). **F** Luciferase activity analyzed after HEK293T cells were co-transfected with the indicated luciferase reporter plasmids, vector, or pcDNA eIF5A2. **G** qRT-PCR results suggesting that eIF5A2 expression in A549 and NCI-H1299 cells decreased after HIF-1α silencing. *P < 0.05, **P < 0.01.

Gene expression profiles change markedly under hypoxia and HIF-1α is a major hypoxia-activated transcription factor. Here, we found that the expression levels of HIF-1α were significantly increased under hypoxic conditions in five NSCLC cell lines (A549, HCC827, NCI-H1703, PC9, and NCI-H1299) (Fig. 2A–D). In addition, we found that eIF5A2 expression was increased by hypoxia in these NSCLC cells (Fig. 2A–D). Interestingly, Spearman correlation analysis based on The Cancer Genome Atlas (TCGA) data revealed that eIF5A2 expression correlated positively with HIF-1α expression (Fig. 2E, R = 0.29, *p* < 0.001). To test whether HIF-1α regulates eIF5A2 expression, luciferase assays were carried out. We constructed luciferase reporter plasmids for the eIF5A2 promotor containing predicted wild-type (WT) and mutant (MUT) HIF-1α binding sites (Fig. 2F), which were co-transfected into HEK293T cells with vector control or a HIF-1α expression vector (pcDNA- HIF-1α). Co-transfection of the eIF5A2-WT and pcDNA- HIF-1α plasmids, but not a negative control, resulted in a significant increase in relative luciferase activity (Fig. 2F). Next, we investigated whether the overexpression of eIF5A2 is mediated through activation of HIF-1α by siRNA-mediated silencing of HIF-1α in these NSCLC cell lines. We found that the expression of eIF5A2 in HIF-1α-silenced A549 and NCI-H1299 cells decreased, indicating that eIF5A2 expression is induced by HIF-1α activation under hypoxic conditions (Fig. 2G). Interestingly, eIF5A2 positively regulates the expression of HIF-1α in esophageal squamous cell carcinoma cell lines [13]. The evidence suggests that eIF5A2 can increase HIF-1α expression and vice versa.

### eIF5A2 is generally overexpressed in NSCLC

**Fig. 3:**
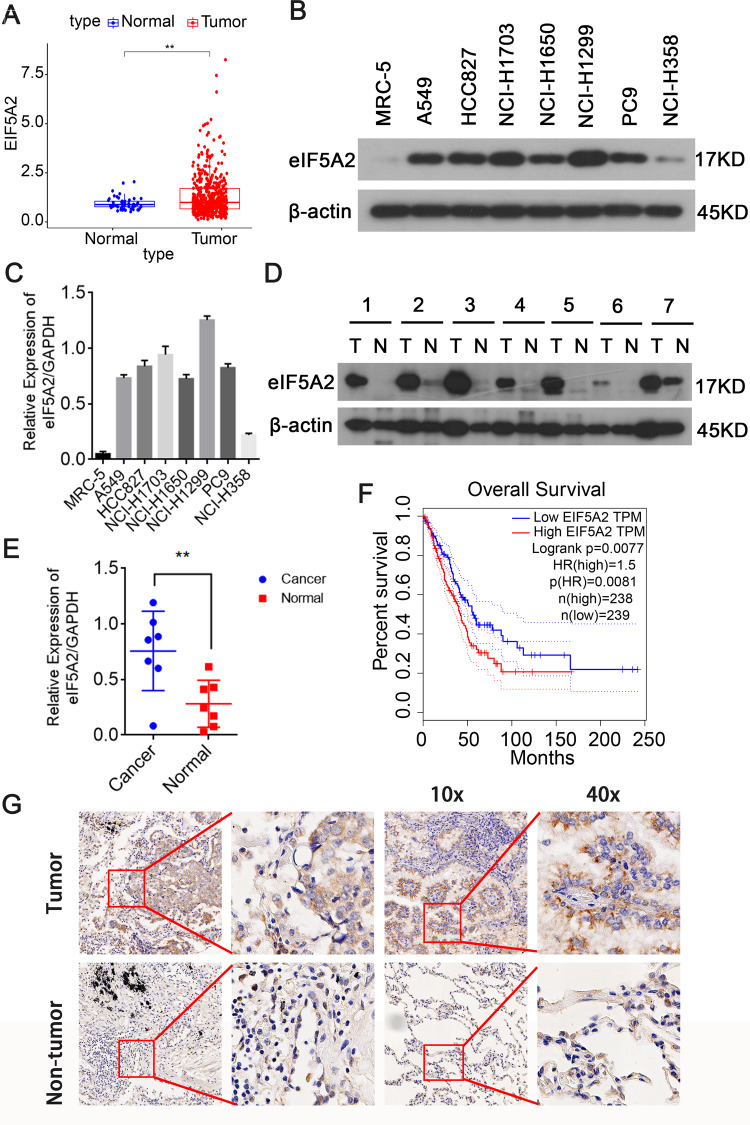
EIF5A2 is highly expressed in NSCLC. **A** Expression analysis based on the TCGA-LUAD cohort showing that eIF5A2 expression in NSCLC tissues was significantly higher than that in adjacent normal tissues. **B**–**E** Western blotting results validating the overexpression of eIF5A2 in both NSCLC cell lines (**B**, **C**) and tissues (**D**, **E**). **F** Survival curve indicating that patients with NSCLC with low eIF5A2 expression always had a better survival outcomes. **G** IHC analysis suggesting that eIF5A2 is highly expressed in NSCLC and is located in the cytoplasm. **P < 0.01.

To further determine the roles of eIF5A2 in NSCLC, were examined eIF5A2 expression levels in NSCLC and normal tissues. Bioinformatic analysis of TCGA data demonstrated that eIF5A2 is overexpressed in LUAD compared with normal tissue (Fig. 3A). We quantified the levels of eIF5A2 in seven NSCLC cell lines and normal lung cells (MRC-5 cells). Except for NCI-H358, eIF5A2 was overexpressed in most NSCLC cell lines (A549, HCC827, NCI-H1703, NCI-H1650, NCI-H1299, and PC9), compared with that in MRC-5 cells (Fig. 3B, C). In addition, eIF5A2 levels were significantly higher in tissues of patients with NSCLC (Fig. 3D, E). Interestingly, survival analysis indicated that patients with NSCLC and low eIF5A2 expression tend to have better survival outcomes (Fig. 3F). Immunohistochemistry (IHC) was used to explore eIF5A2 levels in tumor and normal tissues of two patients with NSCLC using an eIF5A2 monoclonal antibody. The results showed high levels of eIF5A2 in NSCLC tissues and a cytoplasmic location, similar to that predicted using the uniprot database [20] (Fig. 3G). Abnormally high eIF5A2 expression in NSCLC suggests its potential as a therapeutic target to treat NSCLC.

### EIF5A2 contributes to hypoxia-mediated cisplatin resistance in NSCLC

**Fig. 4:**
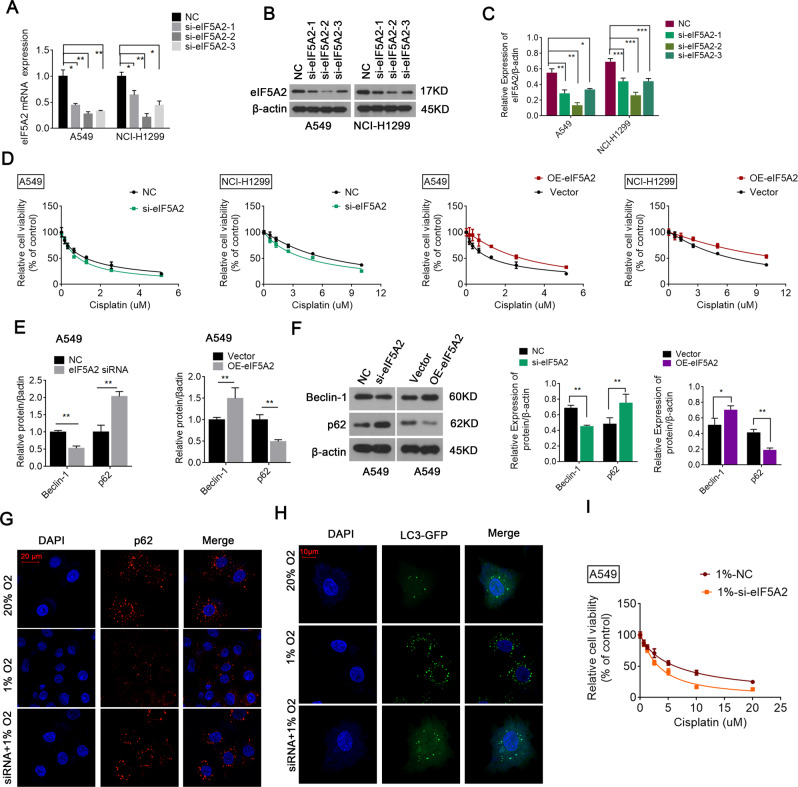
Silencing eIF5A2 effectively decreases the autophagy and cisplatin resistance in NSCLC cells. **A**–**C** qRT-PCR **A** and western blotting **B**, **C** results confirming successful transfection. **D** The results of CCK8 assays suggesting that compared with the negative control, the cell viability of A549 and NCI-H1299 treated with cisplatin decreased after knockdown of eIF5A2, and increased after overexpression of eIF5A2. **E**, **F** qRT-PCR **E** and western blotting (**F**) results proving that compared with the negative control, Beclin-1 expression decreased and p62 expression increased after *EIF5A2* silencing (**E**), which could be reversed by overexpressing eIF5A2 (**F**). **G**, **H** Staining for p62 (green) and DAPI nuclear staining (blue) in A549 cells, confirming the reduced expression of p62 and the enhanced expression of LC3 under hypoxic conditions, and both p62 and LC3 were located in the cytoplasm. However, after silencing eIF5A2, the expression levels of p62 (**G**) and LC3 (**H**) did not decrease, even under hypoxic conditions. **I** The results of a CCK8 assay suggesting that compared with NC-transfected A549 cells, the cell viability of si-eIF5A2-transfected A549 cells treated with cisplatin under hypoxia decreased significantly. *P < 0.05, **P < 0.01.

The high expression of eIF5A2 under hypoxia and in NSCLC suggested that it might contribute to cisplatin resistance in NSCLC. To test this, we reduced eIF5A2 expression using siRNAs targeting eIF5A2. All three siRNAs significantly reduced the mRNA and protein levels of eIF5A2, with si-eIF5A2 presenting the greatest silencing effects (Fig. 4A–C). CCK8 assays showed that compared with the negative control, cisplatin-induced death of A549 and NCI-H1299 cells decreased significantly after eIF5A2 knockdown, and increased after eIF5A2 overexpression (Fig. 4D), indicating that eIF5A2 is involved in cell processes related to cisplatin resistance in NSCLC. Next, we examined the roles of eIF5A2 in hypoxia-induced cisplatin resistance. Compared with the negative control, the viability of si-eIF5A2 transfected A549 cells treated with cisplatin under hypoxia decreased significantly, suggesting that silencing eIF5A2 effectively alleviated hypoxia-induced cisplatin resistance (Fig. 4I).

### EIF5A2 is involved in autophagy in NSCLC cells

Hypoxia-mediated autophagy has been suggested to protect cancer cells from chemotherapy. Therefore, we investigated the roles of eIF5A2 in NSCLC cell autophagy. We found that Beclin-1 expression decreased and p62 expression increased in eIF5A2-silenced A549 cells (Fig. 4E, F). Compared with vector-transfected cells, eIF5A2 overexpressing cells showed higher Beclin-1 expression and lower p62 expression, indicating that eIF5A2 was crucial for enhanced autophagy (Fig. 4E, F). Moreover, rescue experiments showed that P62 siRNA reversed the effect of eIF5A2 siRNA on cisplatin sensitivity (Fig. S1). Immunofluorescence showed that p62 expression levels were reduced (Fig. 4G) and LC3 levels were increased (Fig. 4H) under hypoxic conditions, and both proteins were located in the cytoplasm. Most importantly, after eIF5A2 silencing, p62 (Fig. 4G) and LC3 (Fig. 4H) expression was recovered under hypoxic conditions, indicating that eIF5A2 is essential to trigger autophagy during hypoxia.

**Fig. 5:**
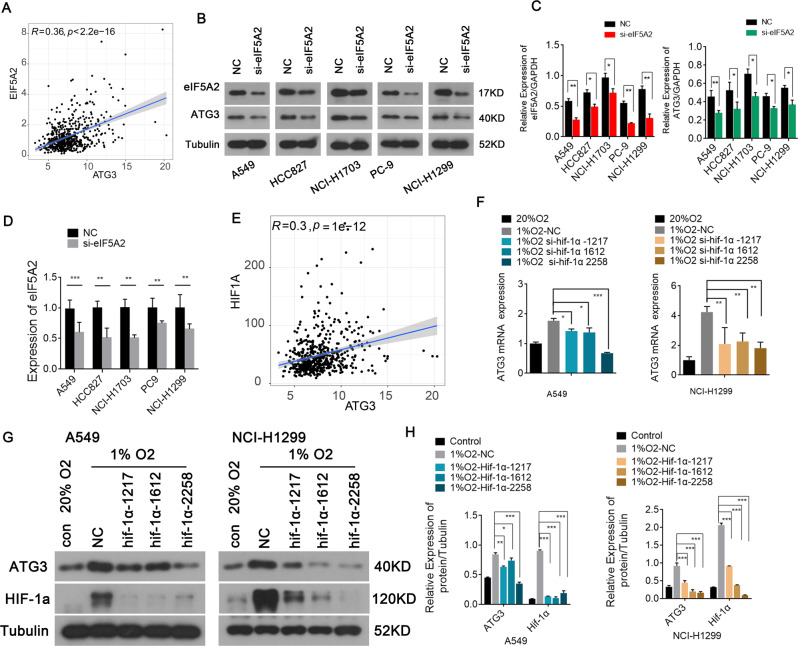
ATG3 could be induced by eIF5A2 and HIF-1α. **A** A scatter plot suggesting that eIF5A2 expression correlated significantly and positively with *ATG3* expression (R = 0.36, *p* < 0.001). **B**, **C** Western blotting results demonstrating that ATG3 levels decreased with the knockout of eIF5A2. **D** qRT-PCR results exhibiting successful transfection. **E** A scatter plot revealing the significant and positive correlation between HIF-1α and *ATG3* expression (R = 0.29, *p* < 0.001). **F**–**H** qRT-PCR (F) and western blotting (**G**, **H**) indicating that ATG3 levels decreased after silencing HIF-1α. *P < 0.05, **P < 0.01, ***P < 0.001.

We next studied another autophagy related gene, ATG3, which is important for autophagosome formation [21]. We calculated the correlation coefficient between HIF-1α and ATG3 expression using Pearson correlation analysis based on TCGA data, which indicated that HIF-1α expression correlated significantly and positively with ATG3 expression (Fig. 5E, R = 0.29, *p* < 0.001). Subsequently, western blotting and quantitative real-time reverse transcription polymerase chain reaction (qRT-PCR) demonstrated the decreased ATG expression when HIF-1α expression levels were reduced (Fig. 5F–H). Next, we analyzed how eIF5A2 affects ATG3 expression. Pearson correlation analysis based on TCGA data indicated that eIF5A2 expression correlates significantly and positively with ATG3 expression (Fig. 5A, *R* = 0.36, *p* < 0.001). Western blotting showed that ATG3 levels in these si-eIF5A2-transfected NSCLC cells (A549, HCC827, NCI-H1703, PC9, and NCI-H1299) were significantly lower than those in the NC-transfected NSCLC cell lines (Fig. 5B, C). qRT-PCR indicated that the ATG3 expression in si-eIF5A2-transfected NSCLC cell lines was markedly inhibited (Fig. 5D). Thus, autophagy in NSCLC is suppressed when eIF5A2 expression is reduced, revealing the crucial role of eIF5A2 in modulating autophagy.

### EIF5A2 downregulation enhances cisplatin’s efficacy against NSCLC tumor growth

**Fig. 6:**
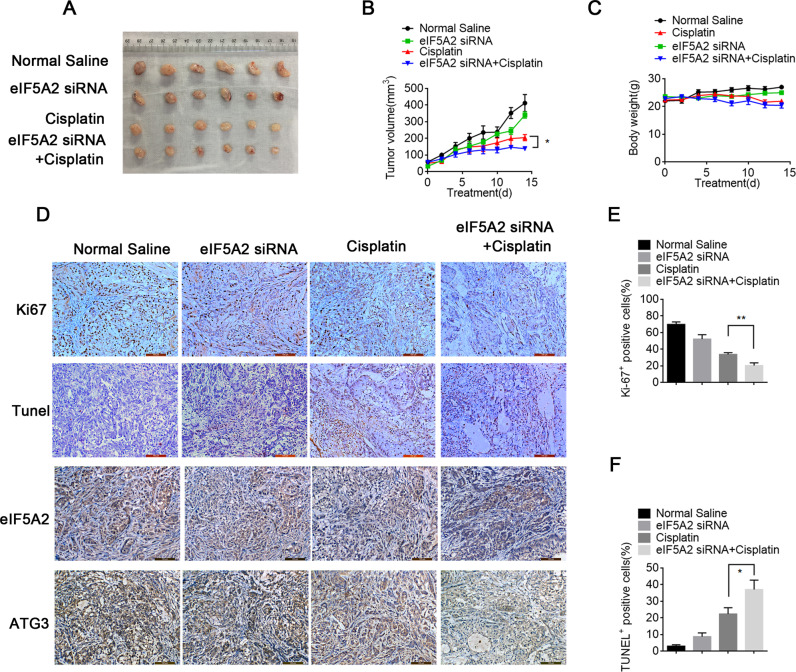
Targeted silencing of eIF5A2 increases cisplatin sensitivity in vivo. **A** The stripped NSCLC tissues were arranged as shown. **B** Tumor volume curve showing that the nude mice silenced for eIF5A2 and treated with cisplatin had the smallest tumor volume, which was statistically significant. **C** There was no statistical difference in the body weight of nude mice among the groups. **D**–**F** According to the results of IHC (**D**), the nude mice silenced for eIF5A2 and treated with cisplatin had the lowest expression of Ki67 (**E**) and the highest level of TUNEL staining (**F**). *P < 0.05, **P < 0.01.

The role of eIF5A2 in cisplatin resistance of NSCLC was further investigated through an in vivo subcutaneous model. The stripped NSCLC tissues are shown in Fig. 6A. We subcutaneously injected A549 cells into nude mice for NSCLC formation, and then injected them with reagents according to their group. Among the four treatment groups (saline, cisplatin only, eIF5A2 silencing only, and cisplatin plus eIF5A2 silencing), the tumors in the nude mice injected with NSCLC tissues with reduced eIF5A2 expression were non-significantly smaller than those in the mice injected with saline. However, cisplatin plus eIF5A2 silencing treatment significantly reduced tumor volume after 15 days compared to the cisplatin alone treated mice, suggesting that eIF5A2 downregulation reinforced the anti-tumor effects of cisplatin. The body weights of the different groups of mice were similar, indicating that the combined treatment had the strongest anti-tumor effects without increasing cisplatin’s toxic side effects (Fig. 6B, C). IHC showed that the mice treated with cisplatin plus eIF5A2 silencing had the lowest Ki67 levels (Fig. 6E) and the highest terminal deoxynulceotidyl transferase nick-end-labeling (TUNEL) staining (Fig. 6F), indicating that the combined treatment effectively reversed hypoxia-induced autophagy and inhibited NSCLC development (Fig. 6D, F and Fig. S2).

## Discussion

Despite its strong nephrotoxicity, cisplatin has remained the cornerstone of NSCLC treatment because of its affordability and effectiveness [22]. However, the emergence of cisplatin resistance has led to therapeutic failure and subsequent poor survival outcomes in patients with NSCLC [23]. The unclear molecular mechanism of chemoresistance is the main obstacle to effective treatment strategies for NSCLC [24]. Therefore, exploring the molecular mechanism of hypoxia-induced cisplatin resistance is important to overcome cisplatin resistance and increase therapeutic efficiency.

In various solid tumors, hypoxia promotes invasiveness, proliferation, and metastasis during tumorigenesis and development, resulting in poor survival outcomes [25]. Crucially, HIF-1α protects cell survival under hypoxic conditions, and is closely related to progression and metastasis of various tumors [25]. IHC analysis of HIF-1α expression in 179 samples of 19 tumor types detected HIF-1α overexpression in 13 tumors, including LUAD [26]. The hypoxic microenvironment caused by high tumor metabolism in various solid tumors leads to increased HIF-1α expression [27]. Herein, we found significantly higher HIF-1α expression under hypoxic conditions than under normoxia condition in NSCLC cells, confirming that HIF-1α overexpression is induced by hypoxia during NSCLC (Fig. 1B, C).

Recently, eIF5A2 overexpression has been reported in a variety of solid tumors, including NSCLC [11], ovarian cancer [28], pancreatic cancer [29], CRC [30], BCA [31], hepatocellular carcinoma [32], and esophageal squamous cell carcinoma (ESCC) [13]. We confirmed high eIF5A2 expression in NSCLC tissues and cell lines. In-depth exploration of the hypoxia mechanism has suggested a correlation between eIF5A2 and HIF-1α. For example, N1-guanyl-1,7-diaminoheptane (GC7), an inhibitor of eIF5A2, reduced doxorubicin resistance in hepatocellular carcinoma cells by reversing hypoxia-induced EMT through the HIF-1α signaling pathway [33]. Herein, we revealed that eIF5A2 expression is induced after hypoxia exposure and is positively regulated by HIF-1α in NSCLC (Fig. 3B–F).

Autophagy is a lysosomal degradation process that digests damaged cell structures, aging organelles, and surplus biological macromolecules, thereby maintaining the homeostasis of microenvironments under harmful conditions, including hypoxia [34]. Recently, the role of hypoxia-induced HIF-1α in modulating autophagy and inducing chemotherapy resistance has been explored. For instance, overexpression of HIF-1α induced by hypoxia increased autophagy in ovarian cancer cells, contributing to cisplatin resistance [35]. Moreover, cisplatin resistance in bladder cancer was induced by activating autophagy under hypoxic conditions, which could be reversed by autophagy or HIF-1α inhibitors [36]. However, whether and how HIF-1α is involved in hypoxia-induced autophagy and chemoresistance in NSCLC cells remains unknown. Our results suggested that HIF-1α plays a crucial part in modulating autophagy by upregulating eIF5A2 in NSCLC cells, leading to cisplatin resistance (Fig. 4D, E).

LC3, p62, Beclin-1, and ATG3 were identified as classical autophagy biomarkers, and their expression represents the level of autophagy indirectly [37]. Various autophagy-related genes (including LC3, p62, Beclin-1, and ATG3) play an essential role in mediating resistance to conventional treatments in NSCLC. For example, a lysosomal inhibitor silencing LC3 decreased the LC3 levels in NSCLC cells, reversing autophagy-modulated icotinib resistance [38]. In addition, p62 induces radiation-resistant cells, suggesting that targeting p62 in patients with NSCLC with a poor radiotherapy response would be an effective treatment strategy [39]. ATG3 is highly expressed in cisplatin resistant NSCLC cells, and downregulation of ATG3 significantly ameliorated cisplatin resistance [40]. Moreover, Beclin-1 has an important role in enhancing chemoresistance in CRC [41]. In this study, we confirmed the involvement of autophagy-related proteins, including LC3, p62, Beclin-1, and ATG3, in the induction of cisplatin resistance in NSCLC cells. Furthermore, reduced eIF5A2 expression downregulated autophagy-related gene expression. In addition, eIF5A2*-*silenced cells showed inhibited hypoxia-induced autophagy, suggesting that eIF5A2 lies upstream of autophagy. These results led us to propose a model for the signaling cascade from hypoxia to cisplatin resistance. In NSCLC, the hypoxic microenvironment activates HIF-1α, which increases eIF5A2 expression. Upregulated eIF5A2 triggers autophagy via an as-yet-unknown mechanism, thereby protecting tumor cells from cisplatin toxicity (Fig. 5F–H).

A major finding of this study is that eIF5A2 silencing drastically reduced cisplatin resistance in NSCLC cells and in vivo models. Hypoxia-induced cisplatin resistance was significantly reduced in eIF5A2-silenced cells under hypoxic conditions (Fig. 4I). In addition, combination therapy comprising eIF5A2 silencing and cisplatin was more effective than cisplatin alone in preventing tumor growth, without increasing cisplatin’s side effects (Fig. 6A–C). The nude mice treated with eIF5A2 silencing and cisplatin had the lowest Ki67 expression and the highest level of TUNEL staining, suggesting that the combined treatment effectively reduced hypoxia-induced autophagy hypoxia and hindered the development of NSCLC (Fig. 6D–F). EIF5A2-targeted inhibition and cisplatin has been found to be effective in other tumors. In a mouse mesenchymal phenotypic oral cancer cell model, silencing eIF5A2 significantly increased the toxicity of cisplatin toward mesenchymal phenotypic oral cancer cells, without an obvious increase in side effects [42].

In order to further explore the detailed mechanism of eIF5A2 regulating autophagy signaling pathway, several experiments were taken. Firstly, we found that eIF5A2 siRNA induced changes in autophagy related indicators, decreased LC3 and Beclin-1 protein and mRNA levels, and increased P62 levels (Fig. 4 and Fig. S2), which was consistent with our previous published study [14]. And further analysis using TCGA database showed that eIF5A2 was positively correlated with ATG3 expression, eIF5A2 siRNA inhibited ATG3 protein levels (Fig. 5). ATG3 is an E2-like enzyme essential for autophagy. Previous studies have shown that ATG3 deletion prevents cells from forming lipidated LC3B, inhibiting membrane elongation and autophagy formation [43, 44]. Lubas et al found that the translation of ATG3 was highly dependent on eIF5A, and the reduced expression of ATG3 caused by eIF5A deletion could not only inhibit the lipatization of LC3B and its adjacent homologues, but also reduce the number of autophagosomes [45]. They also proposed that eIF5A, through its hypine residues, helps ribosomes translate ATG3 protein at its DDG moiety, thus enhancing ATG3 expression and promoting LC3B lipidation and autophagosome formation [45]. EIF5A includes two subtypes, eIF5A1 and eIF5A2. They are the only two proteins that contain the amino acid hypusine [46, 47]. Hypusine modification is critical requirement for eIF5A activity [47]. Therefore, we speculated that eIF5A2 might increase the expression of ATG3 through hypusine modification to promote autophagy, but this needs to be further studied.

## Conclusion

We clarified the signaling cascade involved in hypoxia-mediated cisplatin resistance in NSCLC and identified eIF5A2 as a new therapeutic target to support cisplatin efficacy. Our findings indicated that hypoxia increases eIF5A2 expression via HIF-1α, and overexpression of eIF5A2 could induce cisplatin resistance by enhancing autophagy. Furthermore, the combination therapy comprising eIF5A2 silencing and cisplatin effectively reduced cisplatin resistance and hindered NSCLC development. This study identified new proteins involved in NSCLC cisplatin resistance and provided new insights into effective chemotherapy to inhibit NSCLC development and improve cisplatin’s toxicity toward NSCLC. In future studies, we will further explore the effects of combinations of HIF-1α inhibitors, eIF5A2 inhibitors, and autophagy inhibitors together with cisplatin in patients with NSCLC.

## Methods

### Cell culture and transfection

Human NSCLC cell lines (A549, NCI-H358, HCC827, NCI-H1299, NCI-H1650, and NCI-H1703), human embryonic lung cells (MRC-5), and PC9 cells were obtained from the Shanghai Cell Bank (https://www.cellbank.org.cn/). MRC-5 cells were incubated in Dulbecco’s modified Eagle’s Medium (DMEM, Gibco, Carlsbad, CA, USA), and A549, NCI-H358, HCC827, NCI-H1299, NCI-H1650, NCI-H1703, and PC9 cells were cultured in Roswell Park Memorial Institute (RPMI)−1640 medium (Gibco). Both DMEM and RPMI-1640 medium were supplemented with 10% fetal bovine serum (FBS; Gibco) and 1% penicillin/streptomycin (Sigma, St. Louis, MO, USA). All cells were cultured in 5% CO_2_ at 37 °C. For transfection, cells were seeded in 6-well plates (2.5 × 10^5^ cells/well) and transfected using Lipofectamine 2000 (Invitrogen, Carlsbad, CA, USA). The reagents, including eIF5A2 siRNAs, the eIF5A2 overexpression vector, and their negative control (si-NC, Vector), HIF-1α siRNA and its negative control, were all bought from GenePharma Corporation (Shanghai, China). Primers and other sequences are shown in Table 1.

**Table 1:** Primers utilized for transfection in this study.

Gene	Primer sequence
si-HIF-1α-1217	forward: 5’-GCCGCUCAAUUUAUGAAUATT-3’
	reverse: 5’-UAUUCAUAAAUUGAGCGGCTT-3’
si-HIF-1α-2258	forward: 5’-CCACCACUGAUGAAUUAAATT-3’
	reverse: 5’-UUUAAUUCAUCAGUGGUGGTT-3’
si-HIF-1α-1612	forward: 5’-GCUGGAGACACAAUCAUAUTT-3’
	reverse: 5’-AUAUGAUUGUGUCUCCAGCTT-3’
si-eIF5A2-132	forward: 5’-GCAGACGAAAUUGAUUUCATT-3’
	reverse: 5’-UGAAAUCAAUUUCGUCUGCTT-3’
si-eIF5A2-251	forward: 5’-GGAGAUGUCAACUUCCAAATT-3’
	reverse: 5’-UUUGGAAGUUGACAUCUCCTT-3’
si-eIF5A2-431	forward: 5’-GCUGACAGAAACUGGUGAATT-3’
	reverse: 5’-UUCACCAGUUUCUGUCAGCTT-3’

### Patients and tissue specimens

Tissue samples, including seven paired NSCLC tissues and adjacent normal tissues, were obtained after thoracic surgery in the First Affiliated Hospital of Zhejiang University School of Medicine. The patients provided informed consent for the research use of their tissues. All human-related procedures we conducted according to the 1964 Helsinki Declaration. All samples were quickly frozen and stored in a −80 °C freezer.

### Cell counting Kit-8 (CCK8)

Cell viability was analyzed using CCK8 assays (Dojindo, Kumamoto, Japan) following the manufacturer’s protocol. A549, NCI-H1299 (both 3 × 10^3^ cells/well) and HCC827 (7 × 10^3^ cells/well) cells were seeded in two 96-well plates with corresponding medium. The next day, A549 and NCI-H1299 cells were cultivated inRPMI-1640 medium containing 10% FBS and treated with 0, 0.625, 1.25, 2.5, 5, 10, or 20 μg/mL cisplatin. HCC827 cells were incubated in DMEM with 10% FBS and 0, 0.156, 0.313, 0.625, 1.25, 2.5, or 5 μg/mL cisplatin. Subsequently, the three types of NSCLC cells were incubated in normoxic incubator (37 °C, 20% O_2_, Thermo, Shanghai, China) and a hypoxic incubator (37 °C, 1% O_2_, Thermo), respectively. After incubation for 48 h, we mixed the CCK8 reagent with cell culture medium at 1:10, and added 100 μL/well of the mixed solution to the 96-well plate containing the treated cells. The absorbance of each well was measured at 450 nm after incubation for different periods (1, 2, or 3 h).

### Western blotting

NSCLC cells (2 × 10^5^ cells/well) were seeded in two 6-well plates with corresponding medium, and incubated in normoxic and hypoxic incubators for 48 hours, respectively. Then, the medium was discarded, and the cells were lysed to extract proteins. A bicinchoninic acid (BCA) protein assay kit (Applygen, Beijing, China) was used to quantify the proteins from the NSCLC cells. Subsequently, protein (20 μg/sample) was separated using sodium dodecyl sulfate-polyacrylamide gel electrophoresis, transferred to polyvinylidene difluoride membranes, and incubated in 5% skim milk for 2 h. Then, the membrane was incubated with primary antibodies at 4 °C overnight: anti-eFI5A2 (1:1000, Abcam, Cambridge, UK, ab126733), anti-HIF-1α (1:1000, CST, Danvers, MA, USA), anti-p62 (1:1000, CSTA), anti-light chain 3 (LC3, 1:1000, CST), anti-Beclin-1 (1:1000, CST), anti-autophagy related 3 (ATG3, 1:1000, CST), anti-Tubulin (1:1000, CST), anti-β-actin (1:1000, CST), and anti-glyceraldehyde-3-phosphate-dehydrogenase (GAPDH, 1:1000, ProteinTech, Chicago, IL, USA). After washing three times, the membrane was incubated with the corresponding secondary antibodies (1:2000, CST) at room temperature for 2 h. GADPH, Tubulin, and β-actin was considered normalization controls. Finally, the immunoreactive bands were detected using electro-chemiluminescence.

### Luciferase assay

HEK-293T cells were inoculated into 24-well plates, and then transfected into pGL3- eIF5A2 wild-type promoter region, pGL3-eIF5A2 mutant promoter region firefly lucifase reporter vector (Promega), and co-transfected into pcDNA-HIF-1α overexpression vector or vector control. X-tremegene ™ HP DNA Transfection Reagent (Roche) was used. After transfection, the cells were incubated for 48 h and the luciferase activity was detected using the dual luciferase reporter gene assay system (Promega) according to the manufacturer’s instructions. The results were normalized with renilla luciferase activity.

### Immunofluorescence (IF)

NSCLC cells (4 × 10^4^ cells/well) were seeded in 6-well plates and incubated in normoxic and hypoxic incubators, respectively for 48 h. After washing three times, the cells were fixed using paraformaldehyde for 1 h, and then blocked using 5% FBS for 1 h. Subsequently, anti-LC3 (1:200, CST) and anti-p62 (1:200, CST primary antibodies were added and incubated overnight at 4 °C. The next day, after incubation with the Alexa-conjugated anti-mouse secondary antibodies (Alexa488, Invitrogen) and Alexa-conjugated anti-rat secondary antibody (Alexa555, Invitrogen) at room temperature for 1 h, the cells were stained using diamidino-2-phenylindole (DAPI, Solarbio, Beijing, China) for 5 min. Subsequently, a confocal microscope was used to observe the cells and obtain data.

### Bioinformatic analysis

The transcriptome profiles of LUAD and adjacent normal tissue and the patients’ corresponding clinical information were obtained from the TCGA (https://portal.gdc.cancer.gov/) database [48]. Wilcoxon signed-rank tests were performed using R-x64-4.0.3, ggpubr and limma packages to explore whether there was statistically significant difference in eIF5A2 expression between LUAD tissues and normal tissues, and a column diagram were generated for visualization. Kaplan–Meier survival analysis was performed to determine the statistical differences in OS between high and low eIF5A2 expression groups, and the median eIF5A2 expression value was used as the cut-off to divide LUAD patients into different groups. Spearman correlation analyse was conducted using the R-x64-4.0.3 ggplot2 package to calculate the correlation coefficients among eIF5A2, HIF-1α, and ATG3.

### Immunohistochemistry

The tissue samples from NSCLC patients were embedded in liquid paraffin and cut into 4 μm sections. The primary antibodies were added at 4 °C overnight: anti-eIF5A2 (1:1000, ab227537, Abcam), anti-Ki67 (1:1000, ab15580, Abcam), anti-TUNEL (1:1000, Roche, Basel, Switzerland), anti-ATG3 (1:1000, ProteinTech). Next, after incubation with the corresponding secondary antibodies (1:2000, CST) at 37 °C for 30 min, the sections were stained with a 3, 3’-diaminobenzidine (DAB) plus kit based on the manufacturer’s protocol.

### Quantitative real-time reverse transcription polymerase chain reaction (qRT-PCR)

Total RNA was isolated from NSCLC cells using Trizon (Invitrogen) according to the manufacturer’s protocol, which was subsequently reverse-transcribed into cDNA using a Prime Script RT kit (Takara, Dalian, China). After staining by SYBR Green (Takara), the cDNA was subjected to qPCR in an ABI 7500 Real-Time PCR System (Applied Biosystems, Foster City, CA, USA). The 2-^△△Ct^ method was performed to calculate relative expression values [49], with ACTB and GADPH as normalization controls. All primers used in the present study were purchased from GeneScript (Nanjing, China) (Table 2).

**Table 2:** Primers utilized for qRT-PCR in this study.

Gene	Primer sequence
HIF-1α	forward 5’-ATCCATGTGACCATGAGGAAATG-3’
	reverse 5’-TCGGCTAGTTAGGGTACACTTC-3’
eIF5A2	forward 5’-TGTCCTTCTACTCACAACATGGA-3’
	reverse 5’-CTCACGAACTTCACCAGTTTCT-3’
ATG3	forward 5’-AACATGGCAATGGGCTAC-3’
	reverse 5’-ATCTGTTTGCACCGCTTATAG-3’
Beclin-1	forward 5’-CTCCCGAGGTGAAGAGCATC-3’
	reverse 5’-AATGGAGCTGTGAGTTCCTGG-3’
p62	forward 5’-GAAGCTGCCTTGTACCCACATC-3’
	reverse 5’-TGTCATAGTTCTTGGTCTGCAGGAG-3’
ACTB	forward 5’-TGGCACCCAGCACAATGAA-3’
	reverse 5’-CTAAGTCATAGTCCGCCTAGAAGCA-3’
GADPH	forward 5’-ATCATCAGCAATGCCTCC-3’
	reverse 5’-TCCTTCCACGATACCAAAG-3’

### In vivo subcutaneous model

Animal research was carried out in compliance with the Guide for the Care and Use of the Animal Ethics Committee of Ningbo University. Fifty nude mice were purchased from Hangzhou Ziyuan Laboratory Animal Technology Co., Ltd. (Hangzhou, China), and then subcutaneously injected with A549 cells for NSCLC formation. After one and a half months, we eliminated nude mice with poor tumor formation and fed the remaining nude mice for another 3 weeks. Saline and cisplatin were injected intraperitoneally, and siRNAs were injected intratumorally. The nude mice were injected with saline, eIF5A2 siRNA, cisplatin combined with eIF5A2 siRNA, and cisplatin only, according to their groups. Tumor volume (0.5 × length × width^2^) and the body weight of each nude mouse were measured every 5 days. The nude mice were sacrificed after 15 days, and NSCLC tissues were stripped for subsequent IHC assays. To evaluate apoptosis and proliferation, we utilized IHC to measure Ki67 [50] levels and terminal deoxynulceotidyl transferase nick-end-labeling (TUNEL) staining [51]. In addition, eIF5A2, and ATG3 levels were evaluated by IHC.

### Statistical analysis

Data were expressed as means ± the standard deviation, and were analyzed using GraphPad Prism 6.0 (GraphPad Inc., La Jolla, CA, USA). Student’s t-test or one-way analysis of variance was performed to determine whether there was statistical significance between different groups. All figures were labeled as follows: ***p < 0.001, **p < 0.01, and *p < 0.05.

## Supplementary information

supplementary file

certificate

supplementary file

aj-checklist

## Data Availability

All data generated or analyzed during this study are included in the published article.

## References

[1] Siegel RL, Miller KD, Fuchs HE, Jemal A (2021). Cancer Statistics, 2021. CA Cancer J Clin.

[2] Chen W, Zheng R, Baade PD, Zhang S, Zeng H, Bray F (2016). Cancer statistics in China, 2015. CA Cancer J Clin.

[3] Shi JF, Wang L, Wu N, Li JL, Hui ZG, Liu SM (2019). Clinical characteristics and medical service utilization of lung cancer in China, 2005-2014: Overall design and results from a multicenter retrospective epidemiologic survey. Lung Cancer.

[4] Robinson AD, Chakravarthi B, Agarwal S, Chandrashekar DS, Davenport ML, Chen G (2021). Collagen modifying enzyme P4HA1 is overexpressed and plays a role in lung adenocarcinoma. Transl Oncol.

[5] Dominici C, Sgarioto N, Yu Z, Sesma-Sanz L, Masson JY, Richard S (2021). Synergistic effects of type I PRMT and PARP inhibitors against non-small cell lung cancer cells. Clin Epigenetics.

[6] Tao L, Shu-Ling W, Jing-Bo H, Ying Z, Rong H, Xiang-Qun L (2020). MiR-451a attenuates doxorubicin resistance in lung cancer via suppressing epithelialmesenchymal transition (EMT) through targeting c-Myc. Biomed Pharmacother.

[7] Gaustad JV, Rofstad EK (2021). Assessment of hypoxic tissue fraction and prediction of survival in cervical carcinoma by dynamic contrast-enhanced MRI. Front Oncol.

[8] Liu Y, Wang X, Li W, Xu Y, Zhuo Y, Li M (2020). Oroxylin A reverses hypoxia-induced cisplatin resistance through inhibiting HIF-1alpha mediated XPC transcription. Oncogene.

[9] Kizaka-Kondoh S, Inoue M, Harada H, Hiraoka M (2003). Tumor hypoxia: a target for selective cancer therapy. Cancer Sci.

[10] Kunz M, Ibrahim SM (2003). Molecular responses to hypoxia in tumor cells. Mol Cancer.

[11] Chen C, Zhang B, Wu S, Song Y, Li J (2018). Knockdown of EIF5A2 inhibits the malignant potential of non-small cell lung cancer cells. Oncol Lett.

[12] Liu Y, Du F, Chen W, Yao M, Lv K, Fu P (2015). EIF5A2 is a novel chemoresistance gene in breast cancer. Breast Cancer.

[13] Li Y, Fu L, Li JB, Qin Y, Zeng TT, Zhou J (2014). Increased expression of EIF5A2, via hypoxia or gene amplification, contributes to metastasis and angiogenesis of esophageal squamous cell carcinoma. Gastroenterology.

[14] Wu Y, Tang Y, Xie S, Zheng X, Zhang S, Mao J (2020). Chimeric peptide supramolecular nanoparticles for plectin-1 targeted miRNA-9 delivery in pancreatic cancer. Theranostics.

[15] Wang X, Jiang R, Cui EH, Feng WM, Guo HH, Gu DH (2016). N1-guanyl-1,7-diaminoheptane enhances the chemosensitivity of NSCLC cells to cetuximab through inhibition of eukaryotic translation initiation factor 5A2 activation. Eur Rev Med Pharm Sci.

[16] Pan Q, Sun L, Zheng D, Li N, Shi H, Song J (2018). MicroRNA-9 enhanced cisplatin sensitivity in nonsmall cell lung cancer cells by regulating eukaryotic translation initiation factor 5A2. Biomed Res Int.

[17] Xu G, Yu H, Shi X, Sun L, Zhou Q, Zheng D (2014). Cisplatin sensitivity is enhanced in non-small cell lung cancer cells by regulating epithelial-mesenchymal transition through inhibition of eukaryotic translation initiation factor 5A2. BMC Pulm Med.

[18] Wu HM, Jiang ZF, Ding PS, Shao LJ, Liu RY (2015). Hypoxia-induced autophagy mediates cisplatin resistance in lung cancer cells. Sci Rep..

[19] Lee JG, Shin JH, Shim HS, Lee CY, Kim DJ, Kim YS (2015). Autophagy contributes to the chemo-resistance of non-small cell lung cancer in hypoxic conditions. Respir Res.

[20] The UniProt C. (2017). UniProt: the universal protein knowledgebase. Nucleic Acids Res..

[21] Fang D, Xie H, Hu T, Shan H, Li M (2021). Binding features and functions of ATG3. Front Cell Dev Biol.

[22] Kildey K, Gandhi NS, Sahin KB, Shah ET, Boittier E, Duijf PHG (2021). Elevating CDCA3 levels in non-small cell lung cancer enhances sensitivity to platinum-based chemotherapy.. Commun Biol.

[23] Wang H, Huang H, Wang L, Liu Y, Wang M, Zhao S (2021). Cancer-associated fibroblasts secreted miR-103a-3p suppresses apoptosis and promotes cisplatin resistance in non-small cell lung cancer. Aging (Albany NY).

[24] Wei L, Jiang J (2021). Targeting the miR-6734-3p/ZEB2 axis hampers development of non-small cell lung cancer (NSCLC) and increases susceptibility of cancer cells to cisplatin treatment. Bioengineered.

[25] Seeber LM, Horree N, Vooijs MA, Heintz AP, van der Wall E, Verheijen RH (2011). The role of hypoxia inducible factor-1alpha in gynecological cancer. Crit Rev Oncol Hematol.

[26] Zhong H, De Marzo AM, Laughner E, Lim M, Hilton DA, Zagzag D (1999). Overexpression of hypoxia-inducible factor 1alpha in common human cancers and their metastases. Cancer Res.

[27] Infantino V, Santarsiero A, Convertini P, Todisco S, Iacobazzi V. Cancer cell metabolism in hypoxia: role of HIF-1 as key regulator and therapeutic target. Int J Mol Sci. 2021;22. 10.3390/ijms22115703 PMC8199012 34071836

[28] Zhao G, Zhang W, Dong P, Watari H, Guo Y, Pfeffer LM (2021). EIF5A2 controls ovarian tumor growth and metastasis by promoting epithelial to mesenchymal transition via the TGFbeta pathway. Cell Biosci.

[29] Xu X, Liu H, Zhang H, Dai W, Guo C, Xie C (2015). Sonic Hedgehog-GLI family zinc finger 1 signaling pathway promotes the growth and migration of pancreatic cancer cells by regulating the transcription of eukaryotic translation initiation factor 5A2. Pancreas.

[30] Zhu W, Cai MY, Tong ZT, Dong SS, Mai SJ, Liao YJ (2012). Overexpression of EIF5A2 promotes colorectal carcinoma cell aggressiveness by upregulating MTA1 through C-myc to induce epithelial-mesenchymaltransition. Gut.

[31] Wei JH, Cao JZ, Zhang D, Liao B, Zhong WM, Lu J (2014). EIF5A2 predicts outcome in localised invasive bladder cancer and promotes bladder cancer cell aggressiveness in vitro and in vivo. Br J Cancer.

[32] Tang DJ, Dong SS, Ma NF, Xie D, Chen L, Fu L (2010). Overexpression of eukaryotic initiation factor 5A2 enhances cell motility and promotes tumor metastasis in hepatocellular carcinoma. Hepatology.

[33] Zhou QY, Tu CY, Shao CX, Wang WK, Zhu JD, Cai Y (2017). GC7 blocks epithelial-mesenchymal transition and reverses hypoxia-induced chemotherapy resistance in hepatocellular carcinoma cells. Am J Transl Res.

[34] Jiang N, He D, Ma Y, Su J, Wu X, Cui S (2021). Force-induced autophagy in periodontal ligament stem cells modulates M1 macrophage polarization via AKT signaling. Front Cell Dev Biol.

[35] Long F, Liu W, Jia P, Wang H, Jiang G, Wang T (2018). HIF-1alpha-induced autophagy contributes to cisplatin resistance in ovarian cancer cells. Pharmazie.

[36] Mao X, Nanzhang, Xiao J, Wu H, Ding K (2021). Hypoxia-induced autophagy enhances cisplatin resistance in human bladder cancer cells by targeting hypoxia-inducible factor-1alpha. J Immunol Res.

[37] Lin S, Wang H, Yang W, Wang A, Geng C (2020). Silencing of long non-coding rna colon cancer-associated transcript 2 inhibits the growth and metastasis of gastric cancer through blocking mTOR signaling. Onco Targets Ther.

[38] Zhang Z, Lian X, Xie W, Quan J, Liao M, Wu Y (2020). Role of PARP1-mediated autophagy in EGFR-TKI resistance in non-small cell lung cancer. Sci Rep..

[39] Liu Z, Huang S (2015). Inhibition of miR-191 contributes to radiation-resistance of two lung cancer cell lines by altering autophagy activity. Cancer Cell Int.

[40] Hua L, Zhu G, Wei J (2018). MicroRNA-1 overexpression increases chemosensitivity of non-small cell lung cancer cells by inhibiting autophagy related 3-mediated autophagy. Cell Biol Int.

[41] Li H, Chen C (2017). Inhibition of autophagy enhances synergistic effects of Salidroside and anti-tumor agents against colorectal cancer. BMC Complement Alter Med.

[42] Fang L, Gao L, Xie L, Xiao G (2018). GC7 enhances cisplatin sensitivity via STAT3 signaling pathway inhibition and eIF5A2 inactivation in mesenchymal phenotype oral cancer cells. Oncol Rep..

[43] Sakoh-Nakatogawa M, Kirisako H, Nakatogawa H, Ohsumi Y (2015). Localization of Atg3 to autophagy-related membranes and its enhancement by the Atg8-family interacting motif to promote expansion of the membranes. FEBS Lett.

[44] Nath S, Dancourt J, Shteyn V, Puente G, Fong WM, Nag S (2014). Lipidation of the LC3/GABARAP family of autophagy proteins relies on a membrane-curvature-sensing domain in Atg3. Nat Cell Biol.

[45] Lubas M, Harder LM, Kumsta C, Tiessen I, Hansen M, Andersen JS, et al. eIF5A is required for autophagy by mediating ATG3 translation. EMBO Rep. 2018;19. 10.15252/embr.201846072 PMC5989740 29712776

[46] Nakanishi S, Cleveland JL (2016). Targeting the polyamine-hypusine circuit for the prevention and treatment of cancer. Amino Acids.

[47] Turpaev KT (2018). Translation Factor eIF5A, Modification with Hypusine and Role in Regulation of Gene Expression. eIF5A as a Target for Pharmacological Interventions. Biochem (Mosc).

[48] Tomczak K, Czerwinska P, Wiznerowicz M (2015). The Cancer Genome Atlas (TCGA): an immeasurable source of knowledge. Contemp Oncol (Pozn).

[49] Livak KJ, Schmittgen TD (2001). Analysis of relative gene expression data using real-time quantitative PCR and the 2(-Delta Delta C(T)) Method. Methods.

[50] Zhu HY, Gao YJ, Wang Y, Liang C, Zhang ZX, Chen Y (2021). LncRNA CRNDE promotes the progression and angiogenesis of pancreatic cancer via miR-451a/CDKN2D axis. Transl Oncol.

[51] Yang X, Chen L, Zhao L, Yang Y, Wang J, Yan L (2021). Cordyceps sinensis-derived fungus Isaria felina ameliorates experimental autoimmune thyroiditis in mice. Biomed Pharmacother.

