# Supplementary material for: Eukaryotic initiation factor 5A2 mediates hypoxia-induced autophagy and cisplatin resistance
Source: Cell Death Dis. 2022 Aug 5;13(8):683. doi: 10.1038/s41419-022-05033-y (PMC9356061; doi:10.1038/s41419-022-05033-y)
Supplement: Supplementary file 1 — supplementary file [file 41419_2022_5033_MOESM1_ESM.docx]

Supplementary files


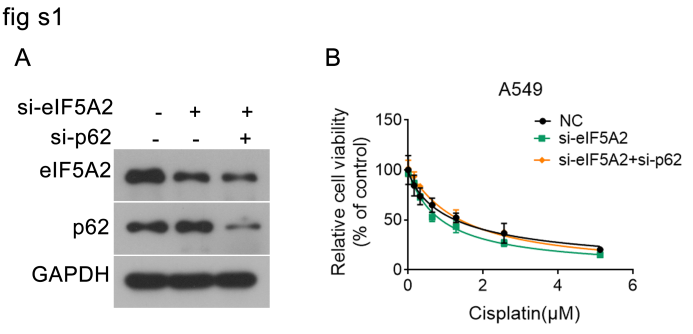


**Figure S1. P62 inhibition reversed the effect of eIF5A2 siRNA on cisplatin sensitivity.** (A) Western blotting results proving that compared with the negative control, p62 expression increased after eIF5A2 silencing, which could be reversed by P62 siRNA. (B) The results of CCK8 assays suggesting that compared with the negative control, the cell viability of A549 treated with cisplatin decreased after knockdown of eIF5A2, which could be reversed by P62 siRNA.


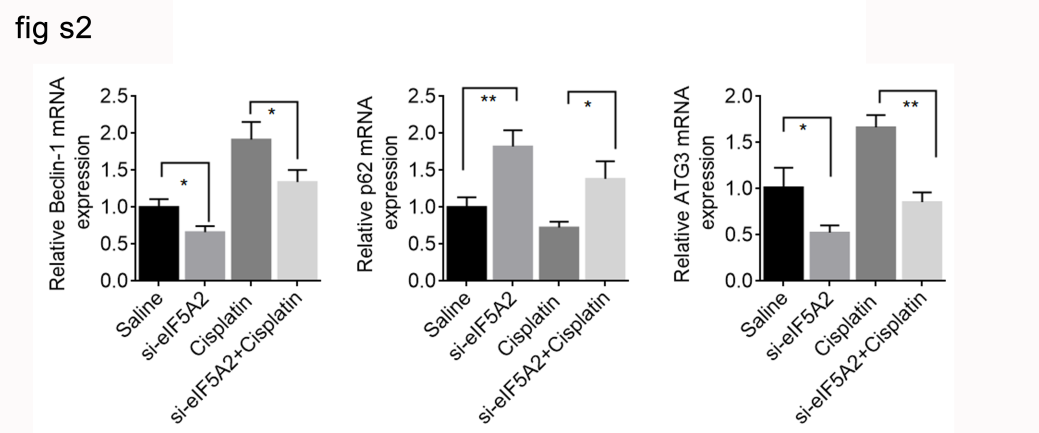


**Figure S2. Beclin-1, and ATG3 were decreased and P62 was increased after si- eIF5A2 treatment in vivo experiment.** *P < 0.05, **P < 0.01
