# Supplementary material for: Eukaryotic initiation factor 5A2 mediates hypoxia-induced autophagy and cisplatin resistance
Source: Cell Death Dis. 2022 Aug 5;13(8):683. doi: 10.1038/s41419-022-05033-y (PMC9356061; doi:10.1038/s41419-022-05033-y)
Supplement: Supplementary file 2 — certificate [file 41419_2022_5033_MOESM2_ESM.pdf]

www.elixigen.com

5/26/2022

Editorial Certification

This document certifies that the manuscript titled "Eukaryotic initiation factor 5A2 mediates hypoxia-induced autophagy and cisplatin resistance" was edited for proper English language, grammar, punctuation, spelling, and overall style by one or more of the highly qualified native English speaking editors at ELIXIGEN.

Neither the research content nor the authors' intentions were altered in any way during the editing process. Documents receiving this certification should be English-ready for publication - however, the author has the ability to accept or reject our suggestions and changes. To verify the final ELIXIGEN edited version, please contact ELIXIGEN at support@elixigen.com.

*We are NOT responsible for any errors in the added content to our revised version after this date.
