# Supplementary material for: Eukaryotic initiation factor 5A2 mediates hypoxia-induced autophagy and cisplatin resistance
Source: Cell Death Dis. 2022 Aug 5;13(8):683. doi: 10.1038/s41419-022-05033-y (PMC9356061; doi:10.1038/s41419-022-05033-y)

Fig1 B

A549-Hif1α
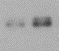
 HCC827-Hif1α
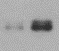
 NCI-H1299-Hif1α
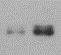


A549-LC3B
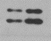
 HCC827-LC3B
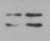
 NCI-H1299-LC3B
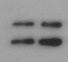


A549-p62
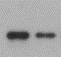
 HCC827-p62
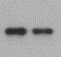
 NCI-H1299-p62
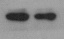


A549-β-actin
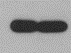
 HCC827-β-actin
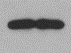
 NCI-H1299-β-actin
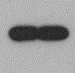


Fig2 A

A549-Hif1α
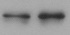
 HCC827-Hif1α
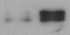
 NCI-H1703-Hif1α
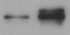
 PC-9-Hif1α
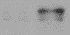


NCI-H1299-Hif1α
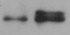


A549 eIF5A2
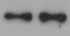
 HCC827 eIF5A2
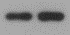
 NCI-H1703 eIF5A2
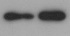
 PC-9 eIF5A2
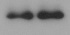
 NCI-H1299 eIF5A2
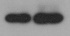


A549-β-actin
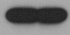
 HCC827-β-actin
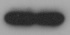
 NCI-H1703-β-actin
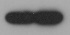
 PC-9-β-actin
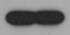
 NCI-H1299-β-actin
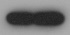


Fig3 B

eIF5A2
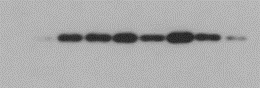


β-actin
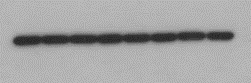


Fig3 D

eIF5A2
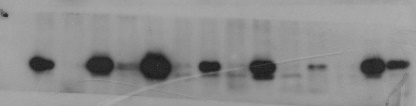


β-actin
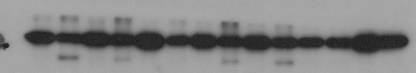


Fig4 B

A549 eIF5A2
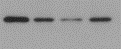
 NCI-H1299 eIF5A2
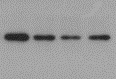


A549-β-actin
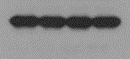
 NCI-H1299-β-actin
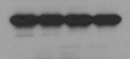


Fig4 F

A549-Beclin-1
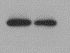
 A549-Beclin-1
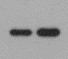


A549-p62
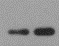
 A549-p62
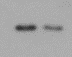


A549-β-actin
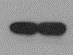
 A549-β-actin
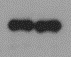


Fig5 B

A549 eIF5A2
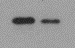
 HCC827 eIF5A2
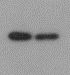
 NCI-H1703 eIF5A2
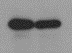
 PC9 eIF5A2
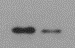
 NCI-H1299 eIF5A2
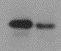


A549 ATG3
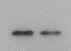
 HCC827 ATG3
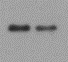
 NCI-H1703 ATG3
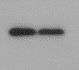
 PC9 ATG3
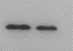
 NCI-H1299 ATG3
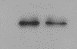


A549 Tubulin
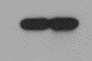
 HCC827 Tubulin
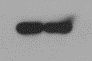
 NCI-H1703 Tubulin
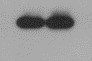


PC9 Tubulin
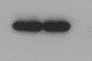
 NCI-H1299 Tubulin
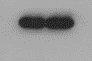


Fig5 G

A549-ATG3
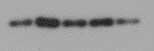
 NCI-H1299-ATG3
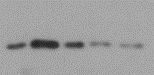


A549- Hif1α
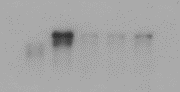
 NCI-H1299-Hif1α
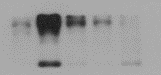


A549- Tubulin
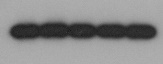
 NCI-H1299-Tubulin
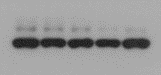

Supplement: Supplementary file 3 — supplementary file [file 41419_2022_5033_MOESM3_ESM.docx]
